# The BnSEP-BnTFL1s module regulates inflorescence architecture based on light duration in *Brassica napus* L

**DOI:** 10.1093/hr/uhaf151

**Published:** 2025-06-09

**Authors:** Lingxiong Zan, Xutao Zhao, Shiying Lv, Haidong Liu, Jingxiu Ye, Yanmei Yao, Kaixiang Li, Dezhi Du

**Affiliations:** Spring Rapeseed Research Institute of Academy of Agricultural and Forestry Sciences, Qinghai University, 251 Ningda Road, Chengbei District, Xining 810016, China; Laboratory for Research and Utilization of Qinghai Tibet Plateau Germplasm Resources, 251 Ningda Road, Chengbei District, Xining 810016, China; Key Laboratory of Spring Rapeseed Genetic Improvement of Qinghai Province, 251 Ningda Road, Chengbei District, Xining 810016, China; Qinghai Spring Rape Engineering Research Center, 251 Ningda Road, Chengbei District, Xining 810016, China; Spring Rapeseed Research Institute of Academy of Agricultural and Forestry Sciences, Qinghai University, 251 Ningda Road, Chengbei District, Xining 810016, China; Laboratory for Research and Utilization of Qinghai Tibet Plateau Germplasm Resources, 251 Ningda Road, Chengbei District, Xining 810016, China; Key Laboratory of Spring Rapeseed Genetic Improvement of Qinghai Province, 251 Ningda Road, Chengbei District, Xining 810016, China; Qinghai Spring Rape Engineering Research Center, 251 Ningda Road, Chengbei District, Xining 810016, China; Spring Rapeseed Research Institute of Academy of Agricultural and Forestry Sciences, Qinghai University, 251 Ningda Road, Chengbei District, Xining 810016, China; Laboratory for Research and Utilization of Qinghai Tibet Plateau Germplasm Resources, 251 Ningda Road, Chengbei District, Xining 810016, China; Key Laboratory of Spring Rapeseed Genetic Improvement of Qinghai Province, 251 Ningda Road, Chengbei District, Xining 810016, China; Qinghai Spring Rape Engineering Research Center, 251 Ningda Road, Chengbei District, Xining 810016, China; Spring Rapeseed Research Institute of Academy of Agricultural and Forestry Sciences, Qinghai University, 251 Ningda Road, Chengbei District, Xining 810016, China; Laboratory for Research and Utilization of Qinghai Tibet Plateau Germplasm Resources, 251 Ningda Road, Chengbei District, Xining 810016, China; Key Laboratory of Spring Rapeseed Genetic Improvement of Qinghai Province, 251 Ningda Road, Chengbei District, Xining 810016, China; Qinghai Spring Rape Engineering Research Center, 251 Ningda Road, Chengbei District, Xining 810016, China; Spring Rapeseed Research Institute of Academy of Agricultural and Forestry Sciences, Qinghai University, 251 Ningda Road, Chengbei District, Xining 810016, China; Laboratory for Research and Utilization of Qinghai Tibet Plateau Germplasm Resources, 251 Ningda Road, Chengbei District, Xining 810016, China; Key Laboratory of Spring Rapeseed Genetic Improvement of Qinghai Province, 251 Ningda Road, Chengbei District, Xining 810016, China; Qinghai Spring Rape Engineering Research Center, 251 Ningda Road, Chengbei District, Xining 810016, China; Spring Rapeseed Research Institute of Academy of Agricultural and Forestry Sciences, Qinghai University, 251 Ningda Road, Chengbei District, Xining 810016, China; Laboratory for Research and Utilization of Qinghai Tibet Plateau Germplasm Resources, 251 Ningda Road, Chengbei District, Xining 810016, China; Key Laboratory of Spring Rapeseed Genetic Improvement of Qinghai Province, 251 Ningda Road, Chengbei District, Xining 810016, China; Qinghai Spring Rape Engineering Research Center, 251 Ningda Road, Chengbei District, Xining 810016, China; Spring Rapeseed Research Institute of Academy of Agricultural and Forestry Sciences, Qinghai University, 251 Ningda Road, Chengbei District, Xining 810016, China; Laboratory for Research and Utilization of Qinghai Tibet Plateau Germplasm Resources, 251 Ningda Road, Chengbei District, Xining 810016, China; Key Laboratory of Spring Rapeseed Genetic Improvement of Qinghai Province, 251 Ningda Road, Chengbei District, Xining 810016, China; Qinghai Spring Rape Engineering Research Center, 251 Ningda Road, Chengbei District, Xining 810016, China; Spring Rapeseed Research Institute of Academy of Agricultural and Forestry Sciences, Qinghai University, 251 Ningda Road, Chengbei District, Xining 810016, China; Laboratory for Research and Utilization of Qinghai Tibet Plateau Germplasm Resources, 251 Ningda Road, Chengbei District, Xining 810016, China; Key Laboratory of Spring Rapeseed Genetic Improvement of Qinghai Province, 251 Ningda Road, Chengbei District, Xining 810016, China; Qinghai Spring Rape Engineering Research Center, 251 Ningda Road, Chengbei District, Xining 810016, China

## Abstract

Rapeseed (*Brassica napus* L.) with determinate inflorescence (DTI) exhibits desirable traits, including reduced plant height, enhanced lodging resistance, and consistent maturity, making them valuable breeding resources. DTI is modulated by *BnaA10.TFL1* and *BnaC09.TFL1* (*BnaA10/C09.TFL1*), which encode the *TERMINAL FLOWER 1* protein, a key regulator of flowering time and meristem identity. However, the underlying functional and regulatory mechanisms remain unclear. In this study, we demonstrated that variations in the promoter region of *BnaA10/C09.TFL1*, rather than the coding region, contributed to the transition from indeterminate inflorescence (IDTI) to DTI in *B. napus*. Specifically, *BnaA10.SEP* inhibited *BnaA10/C09.TFL1* expression by binding to the GT1-motif in the promoter region of *BnaA10/C09.TFL1*^***DTI***^, contributing to the IDTI phenotype under short-day conditions. Meanwhile, two novel DTI mutants were successfully generated through the simultaneous knockout of *BnaA10/C09.TFL1* using the CRISPR/Cas9 system. Furthermore, BnaA10/C09.TFL1 and its homolog BnaA02.FT interacted with BnaA07.14-3-3 instead of directly binding to BnaA08.FD to regulate the development of different inflorescence architectures. Overall, the BnaA10.SEP–BnaA10/C09.TFL1–BnaA07.14-3-3–BnaA08.FD module revealed a new mechanism for DTI formation and a promising strategy for modifying inflorescence architecture traits in *B. napus*.

## Introduction

The inflorescence architecture of higher plants refers to the arrangement of flowers along the inflorescence axis and is predominantly divided into indeterminate inflorescence (IDTI) and determinate inflorescence (DTI). In IDTI, the inflorescence meristem remains active, continuously initiating new floral meristems (FMs), which allows the inflorescences to grow indefinitely. Common examples of plants with IDTI include *Arabidopsis thaliana* and rice. In contrast, DTI involves the conversion of apical meristems into floral meristematic tissue, thereby halting apical growth once a defined number of flowers has been produced, thereby restricting further inflorescence development. Common examples of plants with DTI include *Nicotiana tabacum*, tomato, and Petunia. Generally, plants with DTI are characterized by early flowering, low plant height, and early maturity, making them valuable for crop genetic improvement [1].

The TERMINAL FLOWER 1 (TFL1) protein and its homologs play an important role in determining inflorescence in many plants [2–4]. TFL1 is a small mobile protein lacking a DNA-binding domain (BD); therefore, it must bind with other transcription factors to exert its function [5]. TFL1 competes with FLOWERING LOCUS T (FT) to bind the bZIP transcription factors FLOWERING LOCUS D (FD) and G Box Factor 14-3-3 (14-3-3) proteins in the shoot apex meristem (SAM). This binding regulates the expression of downstream FM recognition genes *LEAFY (LFY)* and *APETALA1 (AP1)* in *Arabidopsis* and rice [3,5]*.* In wild-type *Arabidopsis*, *TFL1* expression inhibits the upregulation of *LFY* and *AP1*. This inhibition prevents the flowering transformation of *Arabidopsis*, delaying flowering, and maintains unlimited plant growth. Moreover, feedback loops from *LFY* and *AP1* can regulate the expression of *TFL1* [6]. However, the absence or downregulation of *TFL1* leads to the binding of FT with FD and promotes the expression of *FLY* and *AP1*. This results in early flowering or the formation of apical flowers in plants, leading to the transformation of IDTI into DTI [7].

Plant flowering and apical meristem development are critical determinants of plant morphogenesis, both of which are finely regulated by light signals. In *Arabidopsis*, flowering is primarily controlled through the GI-CO-FT photoperiod pathway [8]. Upon receiving photoperiod signals, phytochromes and cryptochromes regulate the expression of the circadian clock gene CONSTANS (*CO*). *CO* directly induces the expression of the downstream gene FLOWERING LOCUS T (*FT*). The FT protein is then transported via the phloem to the shoot apex,where it interacts with the FD protein to activate the FM identity gene *AP1*. This activation promotes the expression of the meristem identity protein LFY, ultimately inducing flowering [9,10]. Moreover, light signals maintain the apical meristem homeostasis through the WUS-CLV (WUSCHEL-CLAVATA) feedback loop. Numerous genes involved in this process determine the final morphological structure of floral organs by influencing the differentiation of the SAM and FM. Notably, variations in these genes can lead to changes in floral organ structure [11]. Furthermore, the MADS-box gene family plays a crucial role in inflorescence development, with their expression coregulated by light signals and other endogenous factors [12].

MADS-box transcription factors play a crucial role in various aspects of plant development, such as SAM differentiation, floral organ characteristics, fruit development, and hormone signaling [13]. SEPALATA (SEP) belongs to MADS-box E-class genes and plays an important role in determining floral organ characteristics in the well-known ABCDE model [14]. Previous phylogenetic analysis has revealed that the SEP subfamily undergoes multiple gene replication events during the evolutionary process. The first duplication event leads to two clades called SEP3 and LOFSEP (SEP1/2/4) [15]. The *Arabidopsis* SEP protein can bind to numerous transcription factors in A, B, C, and D classes in flowering models and simultaneously regulate the development of floral organs [16]. For example, SEP4 can interact with SOC1 and AP1/FUL proteins, whereas SEP3 can interact with AP1/FUL, SOC1, AGL6-Like, and CLF and bind to auxin-responsive factors to control flower bud differentiation [17].

Rapeseed (*Brassica napus* L.), a key global source of vegetable oil, is an essential raw material for edible and industrial applications that is widely cultivated in China, Canada, Europe, Australia, and South America [18]. However, wild-type *B. napus* exhibits IDTI characteristics, which lead to many production disadvantages, including taller plants, a tendency for lodging, and inconsistent uniformity and maturity. These traits compromise the efficiency of mechanized harvesting and increase the overall cost of rapeseed cultivation. In our previous studies, we reported that DTI trait in *B. napus* can reduce plant height, improve lodging resistance, promote consistent maturity, and have no negative impact on yield, making them beneficial for mechanized harvesting and helping overcome the limitations associated with IDTI traits [18,19]. Therefore, elucidating the molecular mechanisms underlying DTI formation has important implications for regulating rapeseed architecture and enhancing its adaptation to mechanized harvesting through molecular breeding.

In addition, we have previously discovered the DTI4769 mutant, which was controlled by two pairs of independent recessive genes (*Bnsdt1* and *Bnsdt2*) in a double haploid population. Map-based cloning identified the homologous *BnaA10.TFL1* and *BnaC09.TFL1* of *TFL1* as candidate genes for *Bnsdt1* and *Bnsdt2*, respectively [18,20]. Sequence analysis further revealed two amino acid substitutions (F46L and L47F) in the coding regions of *BnaA10.TFL1* and *BnaC09.TFL1* (*BnaA10/C09.TFL1*) between the IDTI and DTI lines [20,21]. This was verified through the *pBnaA10.TFL1^IDTI^:BnaA10.TFL1^IDTI^* and *pBnaC09.TFL1^IDTI^:BnaC09.TFL1^IDTI^* transgenic rapeseeds [21,22]. However, the underlying functional and regulatory mechanisms of *BnaA10/C09.TFL1* remain unclear.

Therefore, in the present study, we aimed to explore the molecular mechanisms of *BnaA10/C09.TFL1* in the formation of the DTI phenotype in *B. napus*. Through sequence alignment of *B. napus* lines and promoter exchange experiments, we demonstrated that variations in the promoter region of *BnaA10/C09.TFL1*, rather than the two variations in the coding region, contribute to the transition from IDTI to DTI phenotype in *B. napus*. Moreover, using comparative experiments of promoter truncation and site-specific mutagenesis of functional elements, we demonstrated that the first 100-bp sequence of the promoter is crucial. We also conducted yeast one-hybrid (Y1H) experiments, and dual-luciferase reporter gene experiments demonstrated that *BnaA10.SEP* can inhibit *BnaA10/C09.TFL1* expression by binding to the GT1-motif in the promoter region of *BnaA10/C09.TFL1^DTI^*, leading to the formation of the IDTI phenotype under short-day conditions. We further revealed that BnaA10/C09.TFL1 and its homolog BnaA02.FT interact with BnaA07.14-3-3 rather than directly bind to BnaA08.FD, thereby regulating the development of different inflorescence architectures*.* Furthermore, we utilized the CRISPR/Cas9 system to successfully generate two novel DTI mutants. This study offers a promising strategy for modifying DTI traits in *B. napus*.

## Results

### Substitution of two amino acids in *BnaA10/C09.TFL1* does not cause trait changes

**Figure 1:**
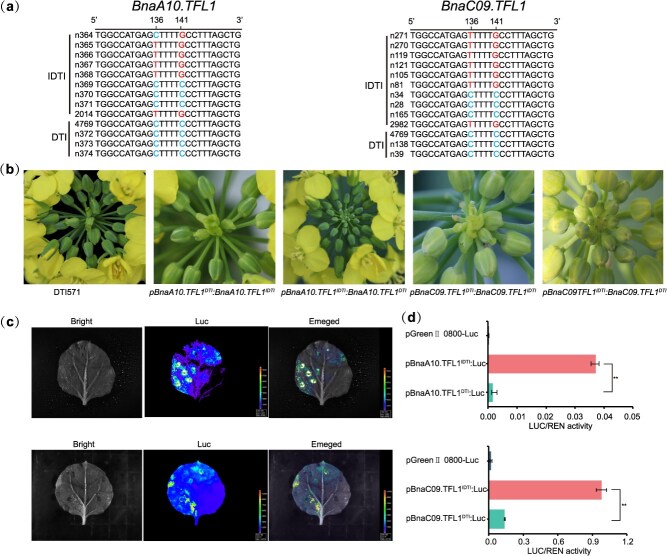
The variation in the promoter region of *BnaA10/C09.TFL1* causes the formation of DTI phenotype. (a) A comparison of the ORF of *BnaA10/C09.TFL1^IDTI^* and *BnaA10/C09.TFL1^DTI^* in various *B. napus* lines. (b) Phenotype of the inflorescence of transgenic positive plants with an exchanged promoter within the *B. napus* DTI571 line. DTI571 and *pBnaA10/C09.TFL1^DTI^:BnaA10/C09.TFL1^IDTI^* exhibit DTI with abnormal floral organs, whereas the IDTI phenotype is recovered in *pBnaA10/C09.TFL1^IDTI^:BnaA10/C09.TFL1^DTI^*. (c) Images of fluorescence signals driven by pBnaA10/C09.TFL1^IDTI^ (left) and pBnaA10/C09.TFL1^DTI^ (top) for luciferase expression in *N. benthamiana* leaves, obtained using a dual-luciferase assay. (d) Quantification of the relative luciferase activity (LUC/REN activity) driven by the *BnaA10/C09.TFL1^IDTI^* and *BnaA10/C09.TFL1^DTI^* promoters in *N. benthamiana* leaves using a dual-luciferase system. Data correspond to the mean ± SD of three biological replicates. Asterisks indicate significant differences (Student’s *t*-test, ^**^P < 0.01).

Our previous studies have demonstrated that the DTI phenotype is a qualitative trait controlled by two recessive loci located on chromosomes A10 and C09, and respectively developed two linked markers for the loci [18,20]. To investigate the function of *BnaA10/C09.TFL1* in inflorescence architecture, we genotyped 374 *B. napus* accessions using these linked markers for the two loci (Table S1). Among these, 5 accessions with DTI and 17 with IDTI were selected for sequence analysis of *BnaA10.TFL1* and *BnaC09.TFL1* (Fig. 1a). DNA sequence analysis revealed two nonsynonymous single-nucleotide polymorphism (SNP) mutations (T136C and G141C) in the exons of *BnaA10/C09.TFL1*, resulting in two amino acid substitutions (Phe46Leu and Leu47Phe). However, these mutations were not associated with the IDTI or DTI phenotypes (Fig. 1a), suggesting that SNP mutations in the coding regions of *BnaA10/C09.TFL1* were not responsible for the transition from IDTI to DTI.

### DNA variations in the *BnaA10/C09.TFL1* promoter is responsible for DTI formation

To investigate functional variations in the promoter regions, we amplified the promoters of *BnaA10/C09.TFL1^IDTI^* and *BnaA10/C09.TFL1^DTI^* from three parental lines used for gene localization: IDTI2014, IDTI2982, and DTI4769, respectively. Sequence comparison of pBnaA10.TFL1^IDTI^ (1845 bp from IDTI2014) and pBnaA10.TFL1^DTI^ (1855 bp from DTI4769) revealed 43 differences, including 32 single-base substitutions and 11 insertion or deletions. We noted no differences between the two promoters at −-715 bp to 0 bp(Fig. S1). However, we identified 68 differences between pBnaC09.TFL1^IDTI^ (1946 bp from IDTI2982) and pBnaC09.TFL1^DTI^ (1908 bp from DTI4769), including 51 single-base substitutions and 17 insertions or deletions (Fig. S2).

To determine if the phenotype change was caused by DNA variations within the *BnaA10/C09.TFL1* promoter, we constructed exchange promoter transgenic *B. napus* DTI line 571 (DTI571). We expressed *BnaA10/C09.TFL1^DTI^* and *BnaA10/C09.TFL1^IDTI^* driven by the promoters of *BnaA10/C09.TFL1^IDTI^* and *BnaA10/C09.TFL1^DTI^* in DTI571, respectively. Compared to DTI571, the *pBnaA10/C09.TFL1^IDTI^:BnaA10/C09.TFL1^DTI^* transgenic plants were restored to the IDTI phenotype, whereas the *pBnaA10/C09.TFL1^DTI^*:*BnaA10/C09.TFL1^IDTI^* transgenic plants exhibited the DTI phenotype (Fig. 1b). To compare the transcriptional activity of the promoters, the fragments of pBnaA10/C09.TFL1^IDTI^ and pBnaA10/C09.TFL1^DTI^ were separately fused with luciferase (LUC) in the vector pGreenII 0800 and expressed in *Nicotiana benthamiana* leaves through transient transformation. The results revealed that the fluorescence signals and LUC/REN activity driven by pBnaA10.TFL1^IDTI^ and pBnaC09.TFL1^IDTI^ were significantly higher than those driven by pBnaA10.TFL1^DTI^ and pBnaC09.TFL1^DTI^ (Fig. 1c and d). Overall, these results indicate that variations in the promoter region of *BnaA10/C09.TFL1* were responsible for the transition from IDTI to DTI phenotypes.

### Mutations in the two SNPs within the *BnaA10/C09.TFL1* promoter leads to a decreased activity

**Figure 2:**
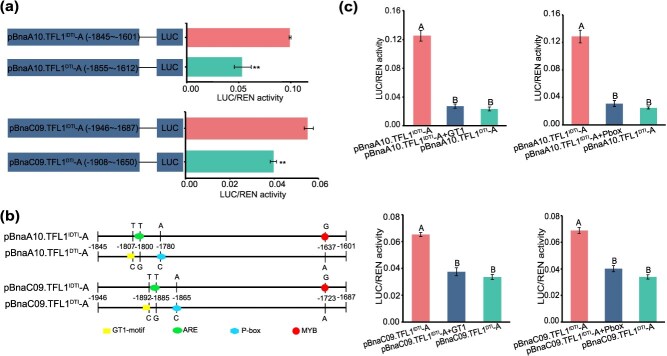
The activity of pBnaA10/C09.TFL1^IDTI^-A with the GT1-motif or P-box elements is significantly reduced, reaching comparable levels to those of pBnaA10/C09.TFL1^DTI^-A. (a) Comparison of LUC/REN activity between constructs driven by pBnaA10/C09.TFL1^IDTI^-A and pBnaA10/C09.TFL1^DTI^-A. (b) Schematic diagram of functional element distribution in key differences (segment A) of the *BnaA10/C09.TFL1* promoter. (c) Quantification of LUC/REN activity after single element site mutation in pBnaA10/C09.TFL1^IDTI^-A compared to that of pBnaA10/C09.TFL1^IDTI^-A and pBnaA10/C09.TFL1^DTI^-A. Data correspond to the mean ± SD of three biological replicates. Different letters indicate significant differences by Tukey’s multiple comparison test with *P* < 0.01. Asterisks indicate significant differences (Student’s *t*-test, ^**^P < 0.01).

To further investigate the promoter of *BnaA10/C09.TFL1*, three truncated fragments (A, B, and C) of different lengths were obtained. These fragments were then fused into the dual-luciferase reporter system (Fig. 2a and Fig. S3a). The LUC/REN activity analysis indicated that the activities of fragments A, corresponding to −1845 to −1601 bp and − 1946 to −1687 bp upstream of the transcription start site (TSS) of *BnaA10.TFL1^IDTI^* and *BnaC09.TFL1^IDTI^* were significantly higher than those of the homologous regions of *BnaA10.TFL1^DTI^* and *BnaC09.TFL1^DTI^*, respectively (Fig. 2a and Fig. S3a). Sequence comparison revealed that the homologous region (fragment A) in *BnaA10/C09.TFL1* possessed four functional elements: the GT1-motif and P-box in pBnaA10/C09.TFL1^DTI^-A, and ARE and MYB in pBnaA10/C09.TFL1^IDTI^-A (Fig. 2b). Moreover, we introduced point mutations in the four elements of pBnaA10/C09.TFL1^IDTI^-A to confirm the core element (Fig. 2c and Fig. S3b). Luciferase analysis revealed that the activity of pBnaA10/C09.TFL1^IDTI^-A with the GT1-motif or P-box elements was significantly reduced to similar levels as those of pBnaA10/C09.TFL1^DTI^-A (Fig. 2c and Fig. S3b). These findings suggest that the two SNP mutations (SNP1-T/C and SNP2-A/C) within the GT1-motif and P-box play an important role in promoter activity.

### Light duration influences DTI phenotypes

**Figure 3:**
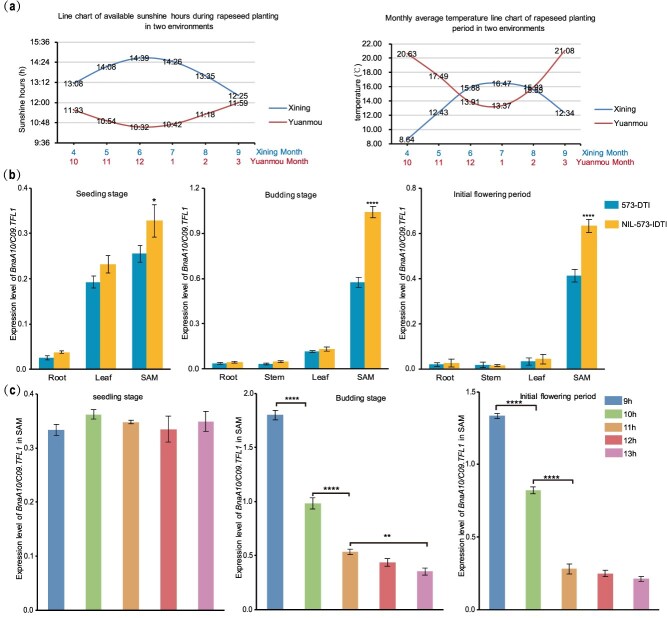
Expression characteristics of *BnaA10/C09.TFL1*. (a) Comparison of sunshine hours and temperature during the rapeseed planting period between Yuanmou (short-day area from October to March) and Xining (long-day area from April to September). (b) Expression analysis of *BnaA10/C09.TFL1* in the root, stem, leaf, and SAM of *B. napus* line 573-DTI and its near-isogenic line NIL-573-IDTI at various growth stages. (c) Expression analysis of *BnaA10/C09.TFL1* in the SAM of 573-DTI under different light durations (9, 10, 11, 12, and 13 h) during the various growth stages. Data correspond to the mean ± SD of three biological replicates. Asterisks indicate significant differences (Student’s *t*-test, ^*^P < 0.05, ^**^P < 0.01, ^***^P < 0.0001).

The late-maturing variety of *B. napus* 573 with DTI in Xining (Qinghai Province, northwest China) exhibited IDTI in Yuanmou (Yunnan Province, southwest China), likely owing to the different climatic environments of Yuanmou (October–March: short-day area) and Xining (April–September: long-day area) (Fig. 3a). Therefore, to investigate the main environmental factors affecting inflorescence architecture, we exposed the 573-DTI line to different light durations, light intensities, and temperatures, respectively. Our results indicated that only light duration affected the inflorescence architecture, with 11 and 9 h emerging as critical light durations for the IDTI and DTI phenotypes, respectively (Fig. S4). Subsequently, we analyzed the expression levels of *BnaA10/C09.TFL1* in the 573-DTI and its near-isogenic line with IDTI (NIL-573-IDTI) using quantitative polymerase chain reaction (qPCR). Among the four different tissues (root, stem, leaf, and SAM), *BnaA10/C09.TFL1* expression was the highest in the SAM. *BnaA10/C09.TFL1* expression was significantly lower in 573-DTI than in NIL-573-IDTI (Fig. 3b). Furthermore, *BnaA10/C09.TFL1* expression in the SAM decreased with increasing light duration from 9 to 13 h (Fig. 3c); however, it was not affected by temperature (Fig. S5a) or light intensity (Fig. S5b). These findings suggest that light duration influences *BnaA10/C09.TFL1* expression, thereby determining the transition between the DTI and IDTI phenotypes.

### Gene editing of *BnaA10/C09.TFL1* to create DTI mutants in *B. napus*

**Figure 4:**
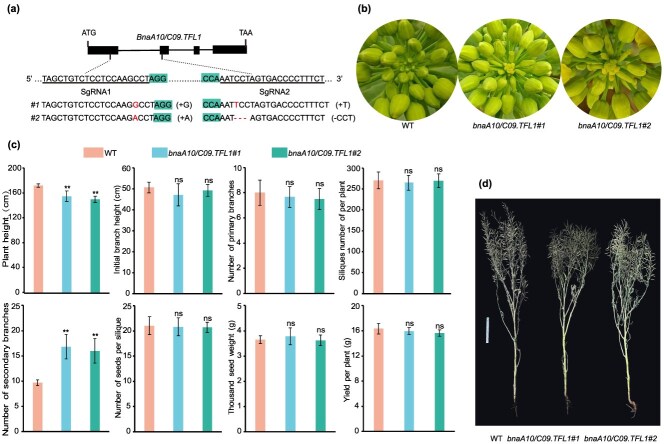
Gene editing of *BnaA10/C09.TFL1* in the Westar variety. (a) Gene editing of *BnaA10/C09.TFL1*. (b) Inflorescence phenotypes of Westar (IDTI) and two transgenic plants (DTI). (c) Comparison of yield-related agronomic traits between the Westar (WT) and two gene knockout lines (*bnaA10/C09.tfl1#1* and *bnaA10/C09.tfl1#2*). Data correspond to the mean ± SD of three biological replicates. Asterisks indicate significant differences (Student’s *t*-test, ^**^P < 0.01). (d) Phenotypes of mature WT and *bnaA10/C09.tfl1* plants. Scale bar = 20 cm.

To investigate whether DTI can occur after complete loss of *BnaA10/C09.TFL1* function, we used the CRISPR/Cas-9 system for gene editing in a *B. napus* line (Westar) with IDTI. Two homozygous mutants (*bnaA10/C09.tfl1#1* and *bnaA10/C09.tfl1#2*) were identified in the T_2_ transgenic plants, both exhibiting the DTI phenotype (Fig. 4a and b). The knockout mutants exhibited lower plant heights, more secondary branches, and no significant alterations in other agronomic traits under field conditions (Fig. 4c and d). Therefore, genome editing of *BnaA10/C09.TFL1* could efficiently decrease plant height without negatively affecting the yield in *B. napus*. Therefore, this mutant can serve as a new germplasm resource.

### *BnaA10/C09.TFL1* expression is regulated directly by *BnaA10.SEP*

**Figure 5:**
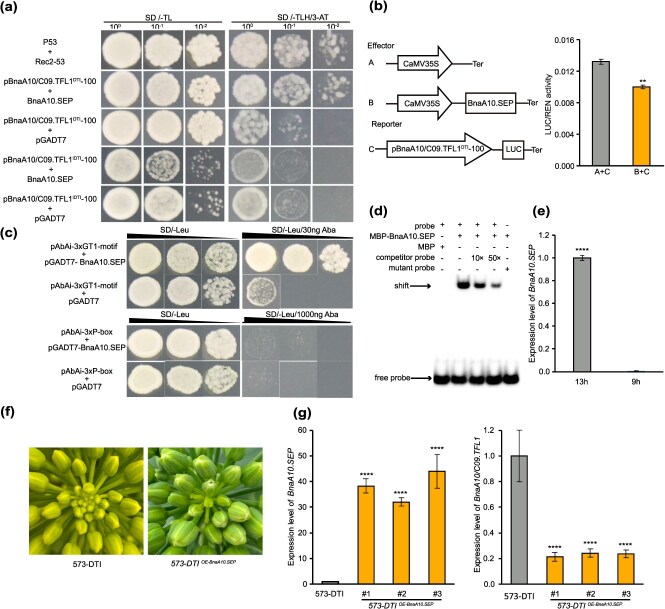
*BnaA10/C09.TFL1* expression is regulated directly by *BnaA10.SEP*. (a) Y1H assays performed using BnaA10.SEP with pBnaA10/C09.TFL1^DTI^-100 and pBnaA10/C09.TFL1^IDTI^-100. (b) A dual-luciferase transient assay indicating that BnaA10.SEP binds to the promoter of *BnaA10/C09.TFL1*^DTI^ and inhibits its expression. (c, d) Y1H assay and EMSA showing that BnaA10.SEP directly binds to the GT1-motif of pBnaA10/C09.TFL1^DTI^-100. MBP, maltose-binding protein. (e) Expression analysis of *BnaA10.SEP* in the SAM during the budding stage of 573-DTI under 13- and 9-h light duration conditions. (f) Phenotypes of 573-DTI and *573-DTI^OE-BnaA10.SEP^* under 9-h light conditions. (g) Expression analysis of *BnaA10.SEP* and *BnaA10/C09.TFL1* in the SAM of the 573-DTI and *573-DTI^OE-BnaA10.SEP^* under 9-h light conditions. Data correspond to the mean ± SD of three biological replicates. Asterisks indicate significant differences (Student’s *t*-test, ^**^P < 0.01, ^***^P < 0.001).

To confirm whether the two SNPs are responsible for the conversion from the DTI to IDTI phenotype under short light durations, Y1H assays were conducted using pBnaA10/C09.TFL1^DTI^-100 (a 100-bp sequence containing both GT1-motif and P-box elements at the beginning of segment A) as bait to screen a complementary DNA (cDNA) library of *B. napus* SAM. Three potential candidate proteins, BnaA10.SEP, BnaA01.ELIP, and BnaC06.FLC, were identified. Subsequent Y1H experiments revealed that BnaA10.SEP can bind to pBnaA10/C09.TFL1^DTI^-100 but not to pBnaA10/C09.TFL1^IDTI^-100 (Fig. 5a and Fig. S6). A dual-luciferase assay confirmed that BnaA10.SEP binds to pBnaA10/C09.TFL1^DTI^-100 and inhibits the transcription of *BnaA10/C09.TFL1^DTI^* (Fig. 5b). Moreover, Y1H and electrophoretic mobility shift assay (EMSA) indicated that BnaA10.SEP specifically bound to the GT1-motif of pBnaA10/C09.TFL1^DTI^-100 (Fig. 5c and d).

Additionally, the expression level of *BnaA10.SEP* in the SAM of 573-DTI was significantly higher under 13-h light duration than under 9-h light duration (Fig. 5e). To verify the regulatory effect of *BnaA10.SEP* on *BnaA10/C09.TFL1*, an overexpression *BnaA10.SEP* line was constructed in 573-DTI and cultured under 9-h light conditions. The results indicated that the overexpression line exhibited the DTI phenotype, whereas the recessive control displayed the IDTI phenotype (Fig. 5f). Moreover, qPCR analysis revealed that the expression level of *BnaA10.SEP* was significantly higher in the overexpression line than recessive control, whereas the expression level of *BnaA10/C09.TFL1* displayed the opposite trend (Fig. 5g). These results indicate that *BnaA10.SEP* negatively regulates *BnaA10/C09.TFL1^DTI^* expression during optimal light exposure, leading to the development of the IDTI phenotype.

### BnaA10/C09.TFL1 physically interact with BnaA07.14-3-3

**Figure 6:**
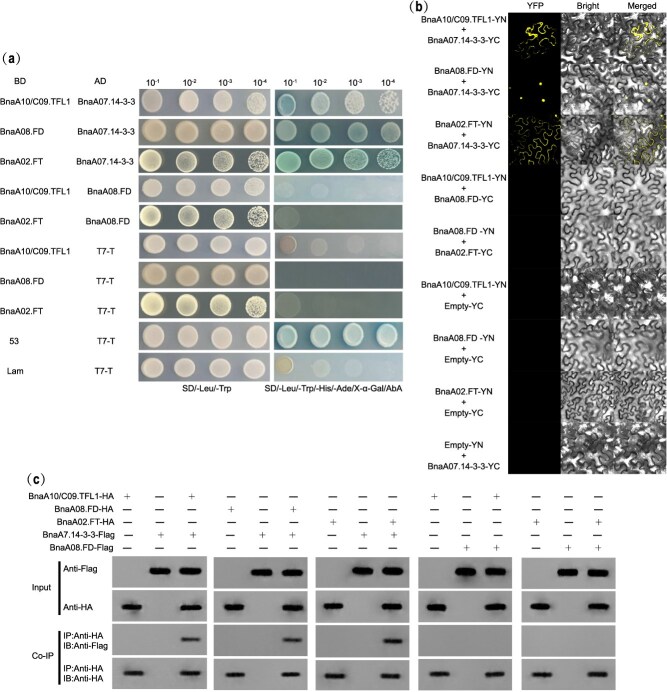
Physical interactions between BnaA10/C09.TFL1 and BnaA02.FT with BnaA07.14-3-3. (a) Yeast two-hybrid (Y2H) assays showing the interactions between BnaA10/C09.TFL1 and BnaA02.FT with BnaA07.14-3-3 and BnaA08.FD. pGBKT7-53/pGADT7-T and pGBKT7-Lam/pGADT7-T served as positive and negative controls, respectively. Values 10^−1^, 10^−2^, 10^−3^, and 10^−4^ indicate the dilution of the yeast cultures prior to being spotted onto the plates. (b) BiFC analysis of the interactions between BnaA10/C09.TFL1 and BnaA02.FT with BnaA07.14-3-3 in the *N. benthamiana* leaves. Scale bars: 50 μm. Images were acquired via a confocal microscope using identical settings. YFP, fluorescent protein; bright, bright field; merge, the figure merged by YFP and bright field. (c) The co-immunoprecipitation (Co-IP) assays indicating the interaction between BnaA10/C09.TFL1 and BnaA02.FT with BnaA07.14-3-3 and BnaA108.FD.

In general, TFL1 does not bind directly to DNA but acts as a transcriptional coregulator by interacting with transcription factors [23]. To further explore the roles of BnaA10/C09.TFL1 in inflorescence formation, a cDNA library from the SAM of *B. napus* was used to identify proteins that interact with BnaA10/C09.TFL1 through yeast two-hybrid (Y2H) assays. Among the potential BnaA10/C09.TFL1-interacting proteins, *A. thaliana* 14-3-3 omega (BnaA07.14-3-3) exhibited a strong interaction with BnaA10/C09.TFL1 (Fig. 6a). In *Arabidopsis*, AtTFL1 typically competes with AtFT to bind to AtFD for function [5]. Our homology comparison revealed that BnaA08.FD and BnaA02.FT shared the highest amino acid identities with *Arabidopsis* AtFD and AtFT proteins, respectively (Fig. S7a and b). In addition, *BnaA08.FD* exhibited the highest expression level among the six BnaFD paralogs (Fig. S7c). Therefore, we focused on analyzing the interaction patterns among four proteins: BnaA10/C09.TFL1, BnaA07.14-3-3, BnaA08.FD, and BnaA02.FT. The Y2H experiments revealed that BnaA10/C09.TFL1 and BnaA02.FT could interact with BnaA07.14-3-3 but not with BnaA08.FD; however, BnaA07.14-3-3 interacted with BnaA08.FD (Fig. 6a). These results were further verified using bimolecular fluorescence complementation (BiFC) assays (Fig. 6b) and co-immunoprecipitation (Co-IP) assays (Fig. 6c). These findings suggest that BnaA10/C09.TFL1 and BnaA02.FT could form protein complexes with BnaA07.14-3-3 and then bind to the BnaA08.FD to exert its function, rather than directly bind to it. This differs from the interaction pattern observed in *Arabidopsis*.

### The BnaA10.SEP–BnaA10/C09.TFL1–BnaA07.14-3-3–BnaA08.FD network regulates inflorescence in *B. napus*

Based on the above experiments, we found that BnaA10.SEP, BnaA10/C09.TFL1, BnaA07.14-3-3, and BnaA08.FD synergistically regulate different inflorescence phenotypes. To further verify this result, we performed two groups of RNA-seq experiments. In the first group, we compared 573-DTI lines under light durations of 9 and 13 h. In the second group, we compared 573-DTI lines with NIL-573-IDTI under 13-h light duration conditions. We identified 4504 differentially expressed genes (DEGs) between the 9 and 13-h light duration conditions and 14 589 DEGs between 573-DTI and NIL-573-IDTI (Tables S2 and S3). We also identified 2761 overlapping DEGs between the two comparative groups and subjected them to Gene Ontology (GO) and Kyoto Encyclopedia of Genes and Genomes *(*KEGG) analysis (Fig. S8a). A relatively large number of DEGs were mainly enriched in multiple regulatory pathways, including signal transduction, specification of floral organ identity, lipid metabolism, and regulation of transcription (Fig. S8b and c; Tables S4 and S5). Furthermore, most key genes regulating flowering time, such as *LFY* and *AP1*, along with flower meristem characteristics, including *AP3* and *PI*, exhibited upregulated expression in both 573-DTI and 573-DTI lines under the 13-h condition compared to NIL-573-IDTI and 573-DTI under the 9-h condition, respectively (Figs S9 and S10). These results suggest that *BnaA10/C09.TFL1* could regulate downstream targets across various regulatory pathways involved in flower development.

**Figure 7:**
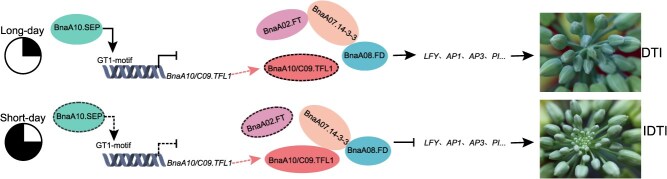
Working model of DTI regulation by *BnaA10.SEP* and *BnaA10/C09.TFL1*. Under long-day conditions, the BnaA10.SEP binds to the GT1-motif of the DTI *BnaA10/C09.TFL1* promoter and inhibits its expression. This causes BnaA02.FT to interact with BnaA07.14-3-3 and subsequently with BnaA08.FD, thus promoting downstream flowering gene expression. Under short-day conditions, low *BnaA10.SEP* expression fails to inhibit *BnaA10/C09.TFL1* or inhibits it only slightly. This allows BnaA10/C09.TFL1 to interact with BnaA07.14-3-3 and then with BnaA08.FD, repressing downstream flowering gene expression and formation of IDTI. Arrows and bars indicate activating and inhibitory effects on genes or proteins. Red dashed lines indicate BnaA10/C09.TFL1 produced by gene expression.

In summary, building on previous studies demonstrating that *BnaA10/C09.TFL1*, homologous genes of *TFL1*, are key genes regulating the inflorescence architecture of *B. napus*, we confirmed that variations in their promoter region contribute to the transition from the IDTI to DTI phenotype in *B. napus*. Specifically, *BnaA10.SEP* inhibits the expression of *BnaA10/C09.TFL1* by binding to the GT1-motif in the promoter region of *BnaA10/C09.TFL1^DTI^*, leading to the IDTI phenotype under short-day conditions. Furthermore, unlike *Arabidopsis*, BnaA10/C09.TFL1 and its homologous BnaA02.FT regulate the development of different inflorescence architectures by interacting with BnaA07.14-3-3 rather than directly binding to BnaA08.FD. Our findings indicate a novel BnaA10.SEP–BnaA10/C09.TFL1–BnaA07.14-3-3–BnaA08.FD network governing inflorescence in *B. napus* (Fig. 7), providing a promising strategy for modifying inflorescence architecture traits in *B. napus.* Therefore, this study has important implications for the future genetic improvement of this important crop.

## Discussion

### T to C substitution at the *BnaA10/C09.TFL1* promoter decreases *BnaA10/C09.TFL1* expression and leads to the DTI phenotype

Most research on DTI traits in *B. napus* involves gene mapping and cloning of candidate genes. For example, Zhang *et al.* found that the DTI locus (dt1) is controlled by *TFL1*, *FVE*, and *SMZ*. Through candidate gene cloning and sequence comparison, they demonstrated that *BnTFL1* has two amino acid differences in DTI and IDTI (Q37 K, G139 S) [24]. Wu reported that *BnTFL1.C9* has an amino acid mutation (V26I) in the DTI mutant SAM3094 compared to IDTI [25]. In our previous study, we demonstrated that *BnaA10/C09.TFL1* has two amino acid differences (F46L and L47F) in the DTI mutant [18,20]. However, the role of these amino acid differences in the transition from IDTI to DTI and the molecular mechanism underlying DTI formation were not explored.

Variations in gene coding regions are recognized as a major cause of gene functional differentiation. However, variations in the regulatory regions of genes also contribute to phenotypic diversity in many plants. For example, SNP-6 in the OsLG1 regulatory region causes a compact panicle structure in rice [26]. Moreover, variations in the fw2.2 regulatory region are associated with changes in tomato fruit size [27]. A 1-bp deletion in the Ms-cd1 promoter leads to dominant male infertility in *Brassica oleracea* [28]. Moreover, a natural variation in an SNP upstream of TGW2 determines grain width and weight in rice [29]. A G/T mutation in the promoter of wild rice GL12 affects its expression, regulating grain length and salt tolerance in cultivated rice [30]. In the present study, a T to C substitution in the promoter region of *BnaA10/C09.TFL1* resulted in *BnaA10/C09.TFL1* inhibition by upstream *BnaA10.SEP.* This resulted in a decrease in expression levels and the formation of the DTI phenotype. Therefore, downregulating the expression of *BnaA10/C09.TFL1* could improve the inflorescence architecture of *B. napus*.

### Light duration regulates DTI phenotype differentiation by affecting the expression of *BnaA10.SEP* and *BnaA10/C09.TFL1*

Environmental factors typically have a greater influence on quantitative traits than qualitative traits. However, our findings revealed that the DTI trait of *B. napus*, despite being a qualitative trait governed by two pairs of genes, exhibits different behavior according to the light duration. This variation is primarily attributed to environmental factors affecting the expression of *TFL1*. Other studies have reported notable effects of light and temperature on *TFL1* expression, although the responses differ among species. For example, in *Arabidopsis*, *TFL1* actively responds to low temperatures by inhibiting plant growth and initiating dormancy, whereas *TFL1* expression is suppressed under high-temperature conditions [31]. In strawberry, *TFL1* is downregulated at low temperatures and upregulated at high temperatures, which prevents flowering [32]. In cucumber, high-temperature signals can lead to the upregulation of *TFL1* and its ortholog *CsTFL1d* [33]. Notably, in the present study, we demonstrated that temperature does not influence the expression of the *BnaA10/C09.TFL1* gene in *B. napus* DTI lines.

In *Arabidopsis* and loquat, *TFL1* expression is inhibited under long-day conditions and enhanced under short-day conditions [31,34]. In cucumber, *TFL1* and its ortholog *CsTFL1d* can be upregulated by long-day signals [33]. In the present study, an increase in light duration promoted the upregulation of *BnaA10.SEP* expression, thereby inhibiting the expression of *BnaA10/C09.TFL1*. This observation also clarifies the inconsistent performance of DTI traits in *B. napus* during southern propagation. In Xining, the sunlight duration during the rapeseed planting period exceeds 12 h (Fig. 3a), enabling DTI lines to exhibit normal DTI traits. In Yuanmou, early-maturing lines typically flower in November, when the sunlight duration is approximately 11 h; thus, DTI lines of *B. napus* can display normal DTI traits during this time. However, late-maturing lines generally flower from late December to early January, when the sunlight duration is less than 11 h. Under short-day conditions, the downregulation of *BnaA10.SEP* expression fails to suppress the expression of *BnaA10/C09.TFL1*, resulting in the upregulation of *BnaA10/C09.TFL1* expression and the IDTI phenotype.

### BnaA10/C09.TFL1 binding to BnaA08.FD requires BnaA07.14-3-3 as a bridge in *B. napus*

In many plants, TFL1 and FT can directly interact with FD to regulate flower meristem characteristic factors such as LFY and AP1 via opposite functions [2]. In *Arabidopsis*, under photoperiod regulation, AtFT and AtTFL1 compete for AtFD and chromatin binding sites, regulating the transition between nutritional and reproductive growth [5]. In potatoes, the reduced expression of *StCEN* accelerates tuber formation, whereas its overexpression delays tuber formation. Notably, StSP6A and StCEN competitively bind to StFD to regulate tuber formation in potatoes [35]. However, in some plants, the interaction between TFL1 and FD requires the involvement of other proteins. For example, in rice, Hd3a cannot directly interact with the OsFD protein, but rather functions through the formation of a ternary transcriptional complex through the 14-3-3 protein [3,36]. In apples, MdFT and MdTFL1 interact with Md14-3-3 protein in the cytoplasm and then translocate to the nucleus, where they bind with MdFD protein to form a complex that jointly regulates the expression of downstream flowering genes [37]. However, in cucumber, CsFT can directly activate the transcription of CsLEY and CsAP1 with CsFD and Cs14-3-3 in the apical meristem, promoting the growth of apical flowers and ultimately limiting overall growth. However, CsTFL1 cannot interact with CsFD and Cs14-3-3 proteins by forming complexes with CsNOT2a and CsFDP, which inhibits the expression of CsLEY and CsAP1, thereby maintaining the unlimited nutritional growth of cucumbers [4]**.**

The interaction mode of TFL1 varies across different plant species. Therefore, to investigate the mechanism of action of BnaA10/C09.TFL1, it is essential to explore their interacting proteins. In the present study, we discovered that both BnaA10/C09.TFL1 and BnaA02.FT interact with the BnaA07.14-3-3, but not with the BnaA08.FD. This finding aligns with a previous report that BnaA10.TFL1 is regulated through interactions between BnaA05.GF14nu and BnaA08.FD proteins [38]. Consequently, our study further confirms that the molecular mechanism of TFL1 in *B. napus* differs from that in *Arabidopsis* and other plants. Specifically, TFL1 must first bind to 14-3-3 before associating with FD to regulate downstream genes associated with FM characteristics.

## Materials and methods

### Plant materials and growth conditions

The DTI material DTI4769 (Bnsdt1Bnsdt1Bnsdt2Bnsdt2), IDTI materials IDTI2982 (BnSDT1BnSDT1BnSDT2BnSDT2) and IDTI2014 (BnSDT1BnSDT1Bnsdt2Bnsdt2), early flowering line DTI571 (carrying two alleles of 4769, Bnsdt1Bnsdt1Bnsdt2Bnsdt2), 573-DTI, NIL-573-IDTI, the Westar line, and a natural population containing 374 accessions of *B. napus* were all provided by the Qinghai Academy of Agriculture and Forestry Sciences, China. All plant materials were grown under standard agronomic conditions, including fertilizers and crop protection chemicals, at the experimental stations. *Nicotiana benthamiana* plants were grown in soil and maintained in growth chambers under long-day conditions (16-h/8-h light/dark conditions) at 22°C.

### Analysis of associations with natural populations and amplification of *BnaA10/C09.TFL1* open reading frames and promoter

The cetyltrimethylammonium bromide method was utilized for DNA extraction from natural populations, following the fourth version of the Molecular Clone protocol. PCR amplification was performed, followed by polyacrylamide gel electrophoresis to analyze the bands. Specific primers were used to amplify the open reading frames (ORF) and promoter regions of *BnaA10.TFL1^IDTI^* and *BnaA10.TFL1^DTI^* in IDTI2014 and DTI4769, as well as *BnaC09.TFL1^IDTI^* and *BnaC09.TFL1^DTI^* in IDTI2982 and DTI4769. The resulting products were cloned into the pMD19-T vector (Takara Bio, Shiga, Japan). At least six clones were subjected to Sanger sequencing, and sequence alignment was performed. Cis-acting regulatory elements were identified using the Plant CARE tool (http://bioinformatics.psb.ugent.be/webtools/plantcare/html/). The sequences of primers used are listed in Table S6.

### Plasmid construction and *Agrobacterium*-mediated genetic transformation

To construct the promoter exchange vector, pBnaA10/C09.TFL1^IDTI^:BnaA10/C09.TFL1^DTI^ and pBnaA10/C09.TFL1^DTI^:BnaA10/C09.TFL1^IDTI^ were cloned into the EcoRI/PstI sites of the pCMBIA2300 vector using whole-genome synthesis. The two vectors were then transformed into DTI571 via *Agrobacterium*-mediated transformation of *B. napus* [39]. To compare the transcriptional activity of the promoters, fragments of pBnaA10.TFL1^IDTI^, pBnaA10.TFL1^DTI^, pBnaC09.TFL1^IDTI^, and pBnaC09.TFL1^DTI^ were individually fused with the linearized vector pGreenII0800-LUC to generate the reporter constructs. The *Renilla* luciferase (REN) gene, driven by the CaMV 35S promoter, was used as an internal control and was expressed in *N. benthamiana* leaves via *Agrobacterium*-mediated transient transformation. *BnaA10/C09.TFL1* knockout vector was generated by designing two guide sequences (sgRNA1 and sgRNA2) within the *BnaA10/C09.TFL1* exon as editing targets for insertion into the zmp1CRISPR/Cas9 vector. The *BnaA10.SEP* coding region (CDS) was amplified using specific primers and inserted into a modified pCAMBIA1300-overexpression plasmid to generate an overexpression vector, which was then transformed into 573-DTI.

### RNA extraction and reverse-transcription qPCR

The 573-DTI line was exposed to various conditions involving different light durations (9, 10, 11, 12, and 13 h), light intensities (4000, 6000, 8000, and 10 000 lx), and temperatures (16°C, 18°C, 20°C, and 22°C), with all other conditions remaining consistent in each test group. Subsequently, SAMs were collected and promptly frozen in liquid nitrogen. Total RNA was extracted using the Plant Total RNA Isolation Kit (no.9769; Takara Bio), following the manufacturer's instructions. RNA concentration was determined using a NanoDrop 2000 spectrophotometer (Thermo Fisher Scientific, Waltham, MA), and RNA quality was assessed via agarose gel electrophoresis. Reverse transcription was performed using the PrimeScript™ RT Reagent Kit with a gDNA Eraser (no. RR047A; Takara Bio). For reverse-transcription qPCR (RT-qPCR), a 10-μl reaction mixture was prepared with 5 μl of 2× TB Green Premix Ex Taq II Mix (Takara Bio), 1 μl of 5× diluted cDNA, 0.2 μl of each primer, and 3.6 μl of ddH2O. The RT-qPCR was conducted using the Light Cycler^®^ 480 Instrument II (Roche, Basel, Switzerland), with *B. napus actin 7* gene serving as the internal control. The relative quantification of gene expression was calculated using the 2^−ΔΔCT^ method [40]. The RT-qPCR results were expressed as the mean ± standard deviation (SD) with three biological and three technical replicates conducted during the experiment.

### Dual-luciferase reporter assay for transactivation analysis

To compare the transcriptional activity of the promoters pBnaA10/C09.TFL1^IDTI^ and pBnaA10/C09.TFL1^DTI^, they were cloned into the vector pGreenII 0800-LUC to generate pBnaA10/C09.TFL1^IDTI^:LUC and pBnaA10/C09.TFL1^DTI^:LUC constructs. A series of truncated fragments of different lengths was fused into pGreenII 0800-LUC. These constructs were used for the transient transformation of *N. benthamiana* leaves. After 2 days, luciferase luminescence was detected using an LB985 NightSHADE system (Berthold Technologies, Belgium, and Germany). The leaves were then ground in liquid nitrogen and dissolved in lysis buffer for luminescence measurements. The firefly luciferase reagent (LARII; 100 μl) was added to the test sample, followed by 20 μl of lysis buffer, and luminescence was measured with a 10-s integration time. Subsequently, 100 μl of the REN reagent and firefly quenching (Stop and Glow™ buffer) were added, and luminescence was measured again with a 10-s integration time. Data are presented as LUC/REN activity, with each data point consisting of at least three biological replicates, and three repeats were performed for each assay.

### Y1H assays

The pBnaA10/C09.TFL1^DTI^-100, TSS upstream of −1855 to −1756 bp for *BnaA10.TFL1^DTI^* or − 1908 to −1809 bp for *BnaC09.TFL1^DTI^*, was cloned into pHIS2 as a bait vector and integrated into the Y187 strain. It was then cotransfected with the *B. napus* cDNA library into the yeast strain for protein screening of pBnaA10/C09.TFL1^DTI^-100 interactions. Three screenings were performed using the lithium acetate conversion method, and the conversion rate was calculated. The conversion solution was evenly spread onto 80 120-mm Triple Dropout Supplements (TDO, SD/−Leu/−Trp/−His/50-mM3-AT) plates, which were then placed upside down in an incubator set at 30°C for 5 days to allow positive clones to grow. These positive clones were transferred to a 0.9% NaCl solution to achieve an OD600 of 0.1. Subsequently, 2.5 μl of each positive clone was inoculated onto SD/−Leu/−Trp/−His/50 mM3-AT plates and cultured at 30°C for 3–5 days to estimate positive clones. Plasmids were extracted from positive clones and sequenced for comparison. Finally, the full length of each transcription factor was individually cloned into the pGADT7 vector to validate the screening results.

### EMSA assay

The *BnaA10.SEP* was amplified and cloned into the pMAL-C5X vector. Recombinant MBP-BnaA10.SEP proteins were expressed in *Escherichia coli* BL21 and purified. A 5′-biotinylated oligonucleotide (5′-tttcaTTAACCtttgggttg-3′) served as the probe. The probes were incubated with nuclear extract at 25°C for 30 min. The reaction mixture was electrophoresed on a nondenaturing 0.5× Tris-Borate-EDTA Buffer 6% polyacrylamide gel for 1 h at 60 V and 4°C, and then transferred onto Biodyne^®^ B nylon membranes (Pall Corporation, Washington, NY). Signal visualization was performed using the reagents provided in the kit on a ChemiDoc XRS system (Bio-Rad Laboratories, Hercules, CA) [41].

### Y2H assays

The CDSs of *BnaA10/C09.TFL1* were amplified and cloned into the Y2H ‘bait’ vector pGBKT7 (Takara Bio). The bait plasmid PGBKT7-BnaA10/C09.TFL1 was introduced into the yeast reporter strain Y2H Gold, along with a *B. napus* SAM cDNA expression library, to identify the proteins interacting with BnaA10/C09.TFL1. Additionally, the CDSs of *BnaA07.14-3-3*, *BnaA08.FD*, and *BnaA02.FT* were amplified using PCR and cloned into the vectors pGADT7 and pGBKT7. The bait and prey constructs were cotransformed into the yeast strain Y2H Gold. Cotransformed yeast clones were serially diluted, spotted onto SD/−Trp/−Leu medium and SD/−Trp/−Leu/−His/−Ade/X-α-Gal/AbA medium, and then cultured at 30°C. The pGBKT7-53/pGADT7-T, pGBKT7-Lam/pGADT7-T, and pGBKT7-BD/pGADT7-T were used as positive control, negative control, and BD self-activation verification, respectively.

### BiFC assay

The CDSs of *BnaA10/C09.TFL1*, *BnaA07.14-3-3*, *BnaA08.FD*, and *BnaA02.FT* were initially cloned into the entry vector pDONR221 using Gateway technology (Invitrogen, Carlsbad, CA). Subsequently, they were transferred to either pEarly Gate201-YN or pEarly Gate202-YC. The resulting constructs were separately transformed into *Agrobacterium tumefaciens* GV3101. The GV3101 cells containing the two vectors were combined at a 1:1 ratio and coinfiltrated into *N. benthamiana* leaves. After 48 h of infiltration, infected leaves were examined for yellow fluorescent protein (YFP) signals using confocal microscopy. This experiment was conducted in triplicate.

### Co-IP assay

Total protein was extracted from agro-infiltrated tobacco leaves in the CoIP assay using a Plant Protein Extraction Kit (ComWin Biotech, Beijing, China). Co-IP assays were conducted using a Pierce™ Magnetic HA-Tag IP/Co-IP Kit (Thermo Fisher Scientific) according to the manufacturer’s instructions. The blots were probed using DyLight 488-conjugated rabbit anti-HA-Tag and Alexa Fluor 647-conjugated mouse anti-Flag-Tag antibodies (Antibodies-Online GmbH Aachen, Germany), following the manufacturer’s protocol.

### RNA-seq analysis

The SAMs from the budding stage of 573-DTI and NIL-573-IDTI at 13 h, and 573-DTI at 9 h of light treatment were collected for RNA extraction. Each sample comprised three individuals with three biological replicates, totaling nine individuals per sample. The RNA libraries were sequenced by OE Biotech, Inc., Shanghai, China, using an Illumina platform (Illumina Inc., San Diego, CA). The generated reads were cleaned and then aligned against the *B. napus* ZS11 reference genome. Fragments per kilobase million (FPKM) read values were used to calculate gene expression levels using feature counts. DEGs were determined using DESeq2 with thresholds of |Log2 (fold-change)| > 1 and a *q* value of <0.05. After z-score normalization, the generated FPKM values were analyzed to assess the transcript abundance. RNA sequencing, data analysis, DEG identification, and GO and KEGG analysis were performed as previously described [42].

### Statistical analysis

Data for statistical analyses were obtained from three biological replicates and analyzed using GraphPad Prism V8.0 (GraphPad Inc., San Diego, CA). Data correspond to the mean ± SD of three biological replicates. Statistically significant differences were calculated using the Student’s *t*-test (^*^P < 0.05, ^**^P < 0.01, ^***^P < 0.001, ^****^P < 0.0001) or one-way analysis of variance with Tukey’s multiple comparison test. Different uppercase letters indicate statistically significant differences between samples (*P* < 0.01); the same uppercase letters indicate insignificant statistical differences between samples.

## Supplementary Material

Web_Material_uhaf151

## Data Availability

All data supporting the findings of this study are available within the paper and its supplementary data published online. Raw RNA-seq reads generated in this study have been deposited into the CNGB Sequence Archive (CNSA) of China National GeneBank Database (CNGBdb; https://db.cngb.org/) with accession numbers CNP0005923.
