# Supplementary material for: The BnSEP-BnTFL1s module regulates inflorescence architecture based on light duration in Brassica napus L
Source: Hortic Res. 2025 Jun 9;12(9):uhaf151. doi: 10.1093/hr/uhaf151 (PMC12373974; doi:10.1093/hr/uhaf151)

**The following supplement information is available for this article:**

**Fig. S1** Sequence comparison of pBnaA10.TFL1^IDTI^ (1,845 bp from IDTI2014) and pBnaA10.TFL1^DTI^ (1,855 bp from DTI4769).

**
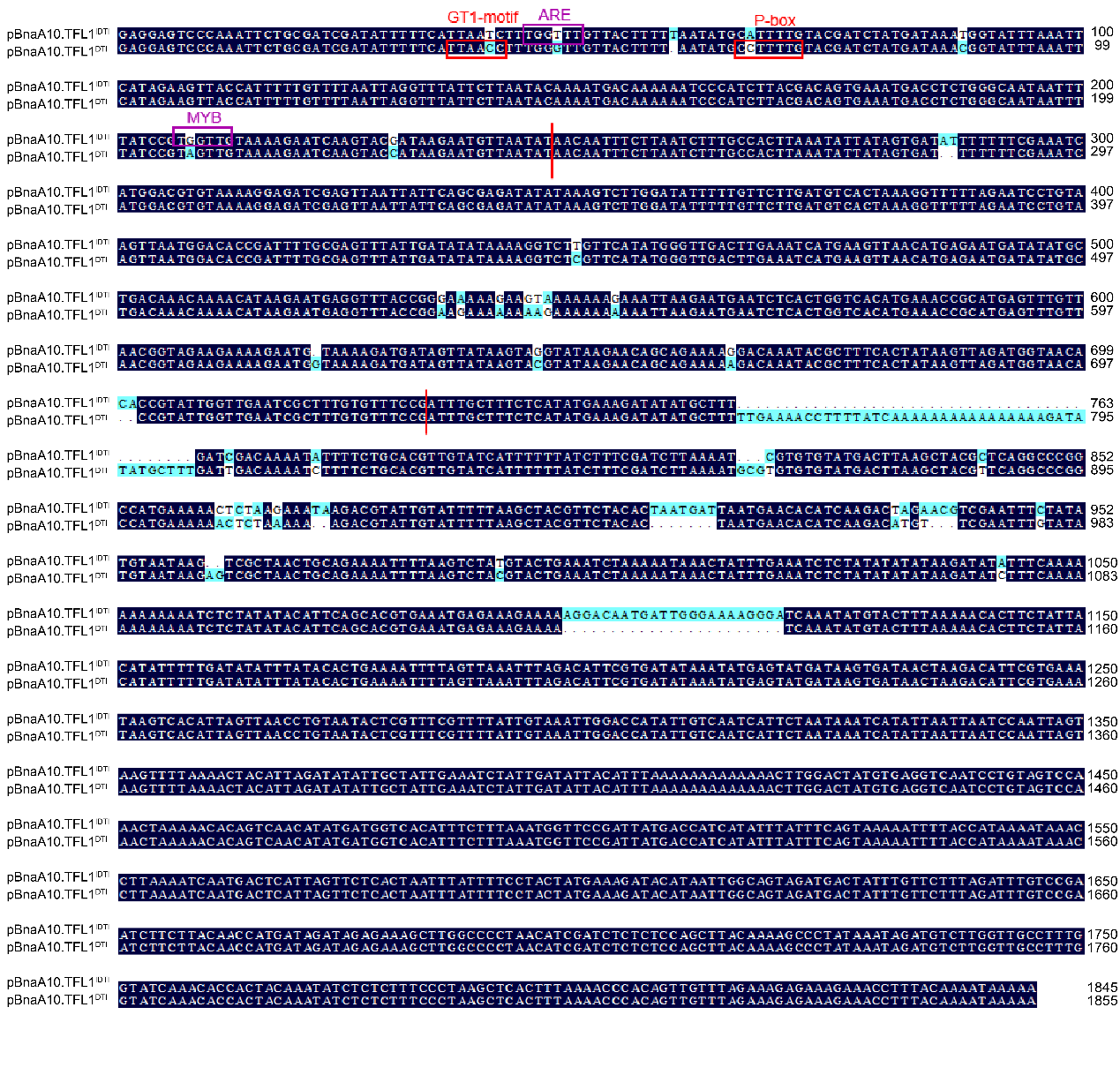
**

**Fig. S2** Sequence comparison of pBnaC09.TFL1^IDTI^ (1,946 bp from IDTI2982) and pBnaC09.TFL1^DTI^ (1,908bp from DTI4769).

**
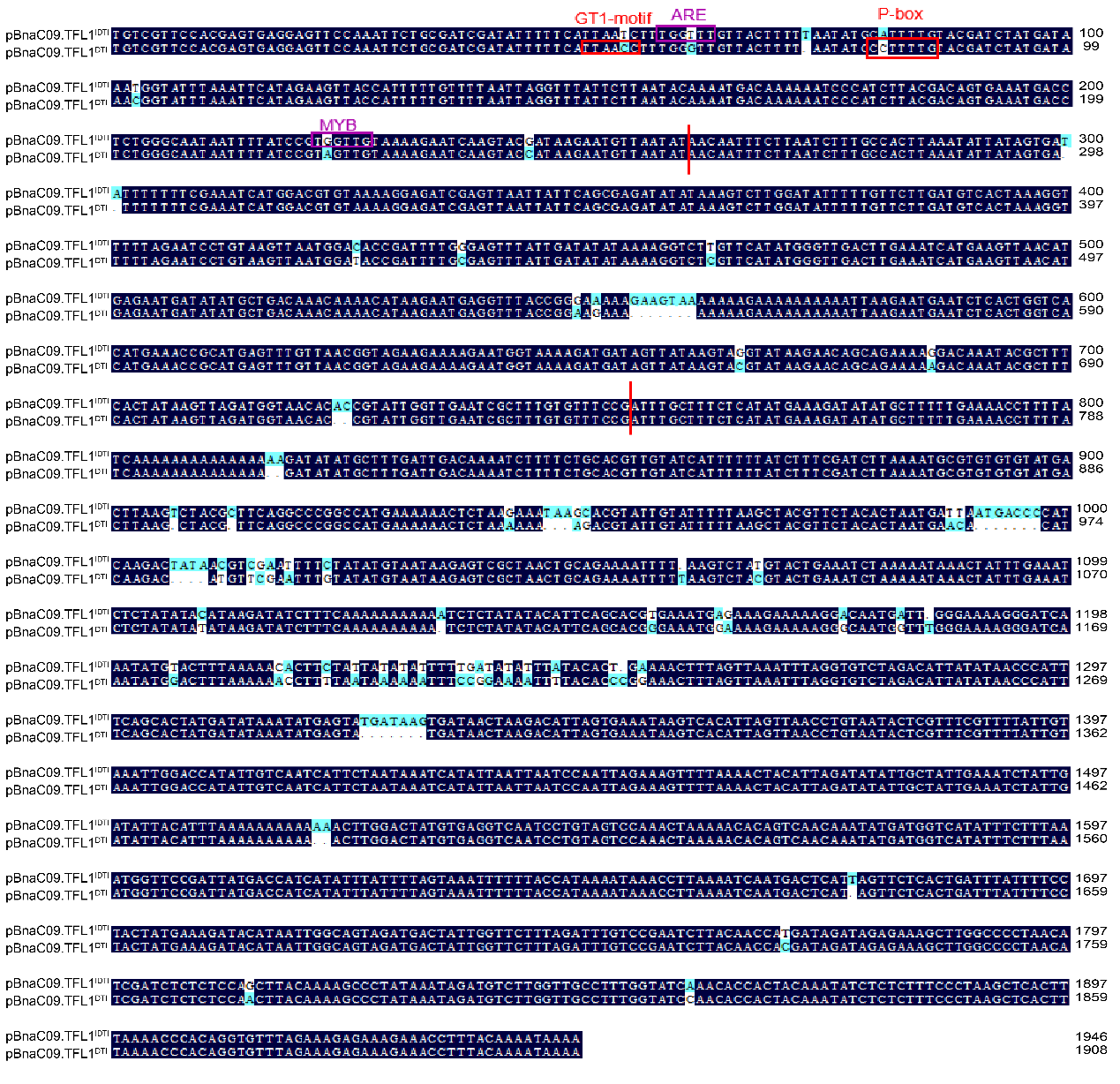
**

**Fig. S3** Segmented analysis of promoters and point mutation of elements in pBnaA10/C09.TFL1-A using the dual-luciferase assay. **(a)** LUC/REN activity was measured in *Nicotiana benthamiana* leaves using a dual-luciferase system with various promoter fragments to drive luciferase expression. **(b)** LUC/REN activity was measured after single element site mutation in pBnaA10/C09.TFL1-A using a dual-luciferase system. The activities of p BnaA10/C09.TFL1^IDTI^-A and p BnaA10/C09.TFL1^DTI^-A were compared.


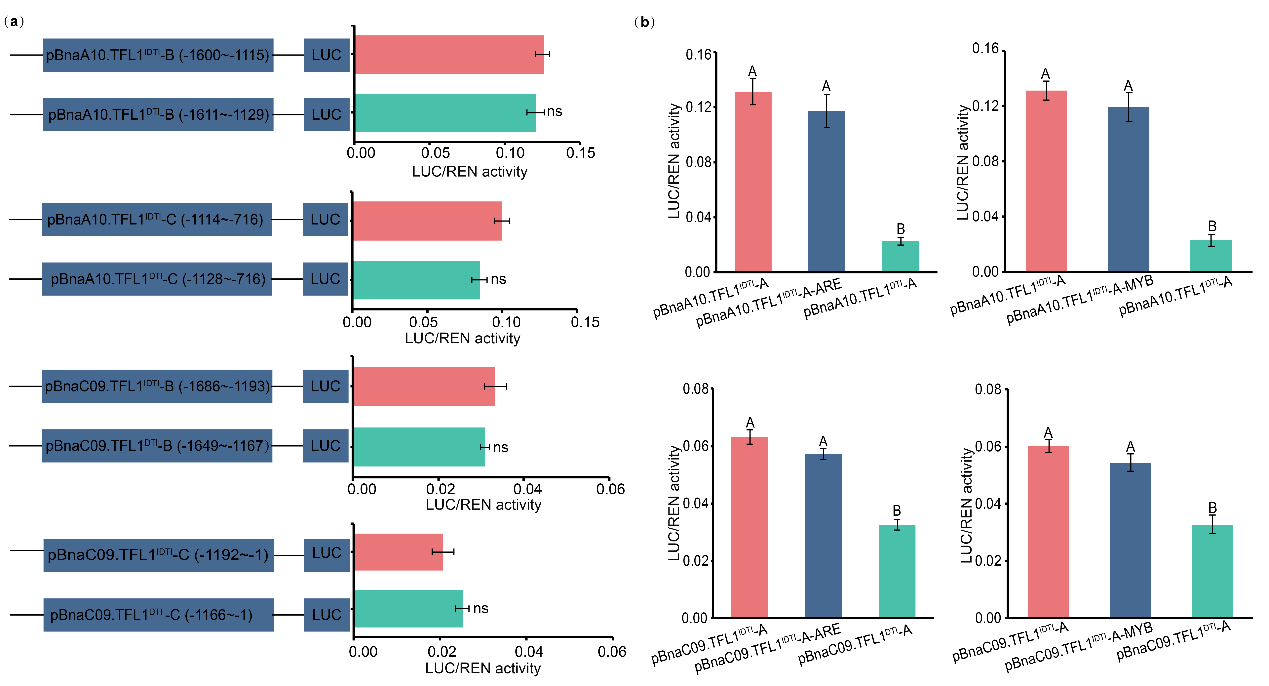


**Fig. S4** Phenotypic characteristics of 573-DTI under different treatments.


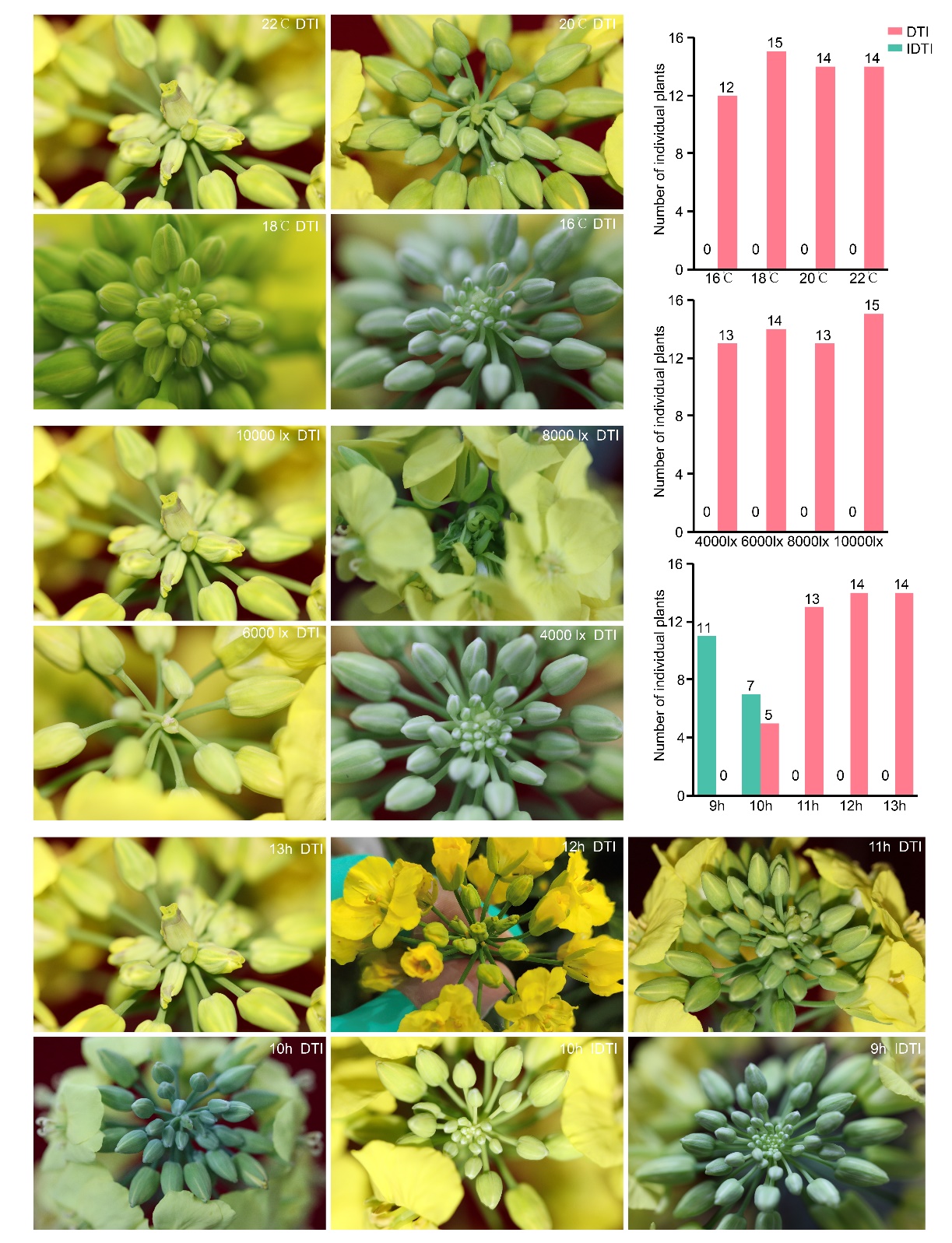


**Fig. S5** Expression characteristics of *BnaA10/C09.TFL1* in the shoot apical meristem (SAM) under different treatments. **(a)** Expression analysis of *BnaA10/C09.TFL1* in the SAM of 573-DTI under various temperature (16, 18, 20, and 22℃) during the different growth stages. **(b)** Expression analysis of *BnaA10/C09.TFL1* in the SAM of 573-DTI under various light intensity treatments (4,000, 6,000, 8,000, and 1,0000 lx) during the different growth stages.


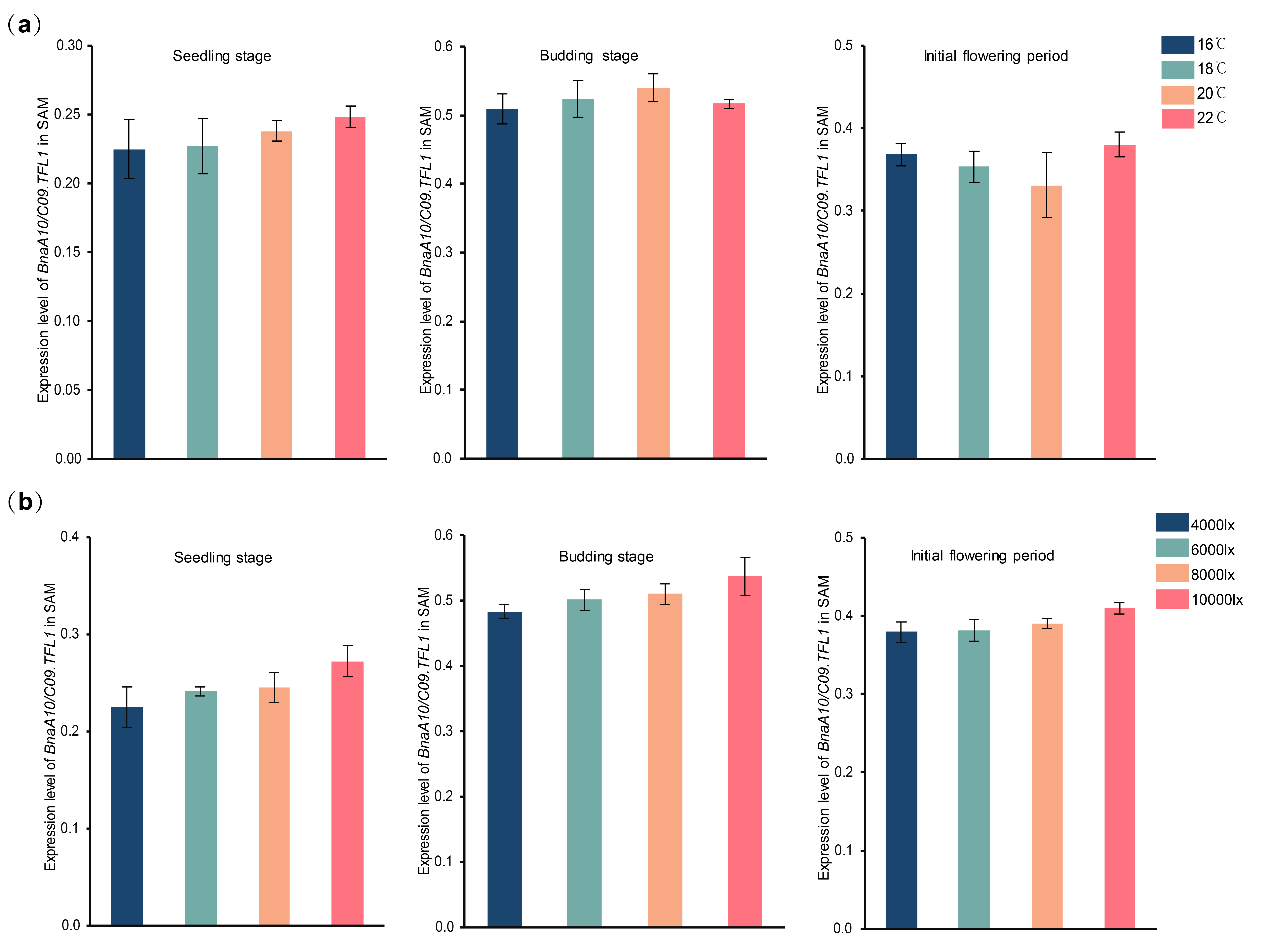


**Fig. S6** Yeast one-hybrid (Y1H) assays. YIH assays were conducted using BnaA01.ELIP and BnaC06.FLC with pBnaA10/C09.TFL1^DTI^-100 and pBnaA10/C09.TFL1^IDTI^-100, respectively. Transformed yeast cells were grown on SD medium lacking Leu and His or SD/-His/-Leu/-Trp containing 3-AT. 10^0^, 10^−1^, and 10^−2^ indicate the dilution of the yeast cultures prior to being spotted onto the plates.


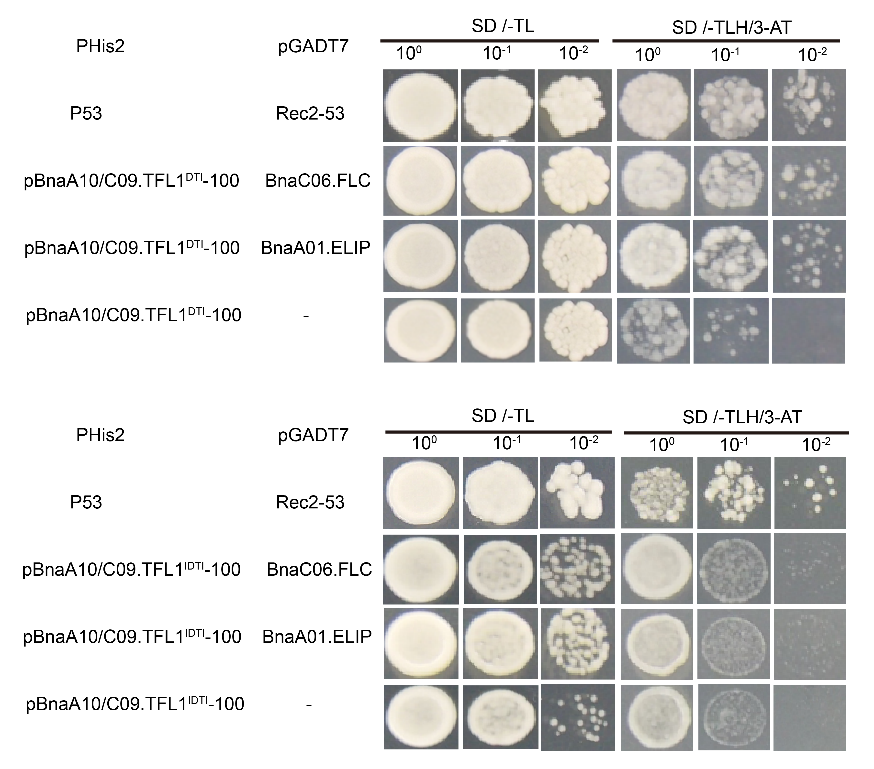


**Fig. S7** Homology analysis of BnaFD and BnaFT in *B. napus* and *Arabidopsis* and expression analysis of *BnaFD*. Sequence identity analysis of full-length amino acid sequences of AtFD (a) and AtFT (b) proteins in *B. napus* (variety 'ZS11') and *Arabidopsis thaliana*. (c) Analysis of the expression patterns of all *BnaFD* genes in *B. napus* (IDTI2982) SAM.


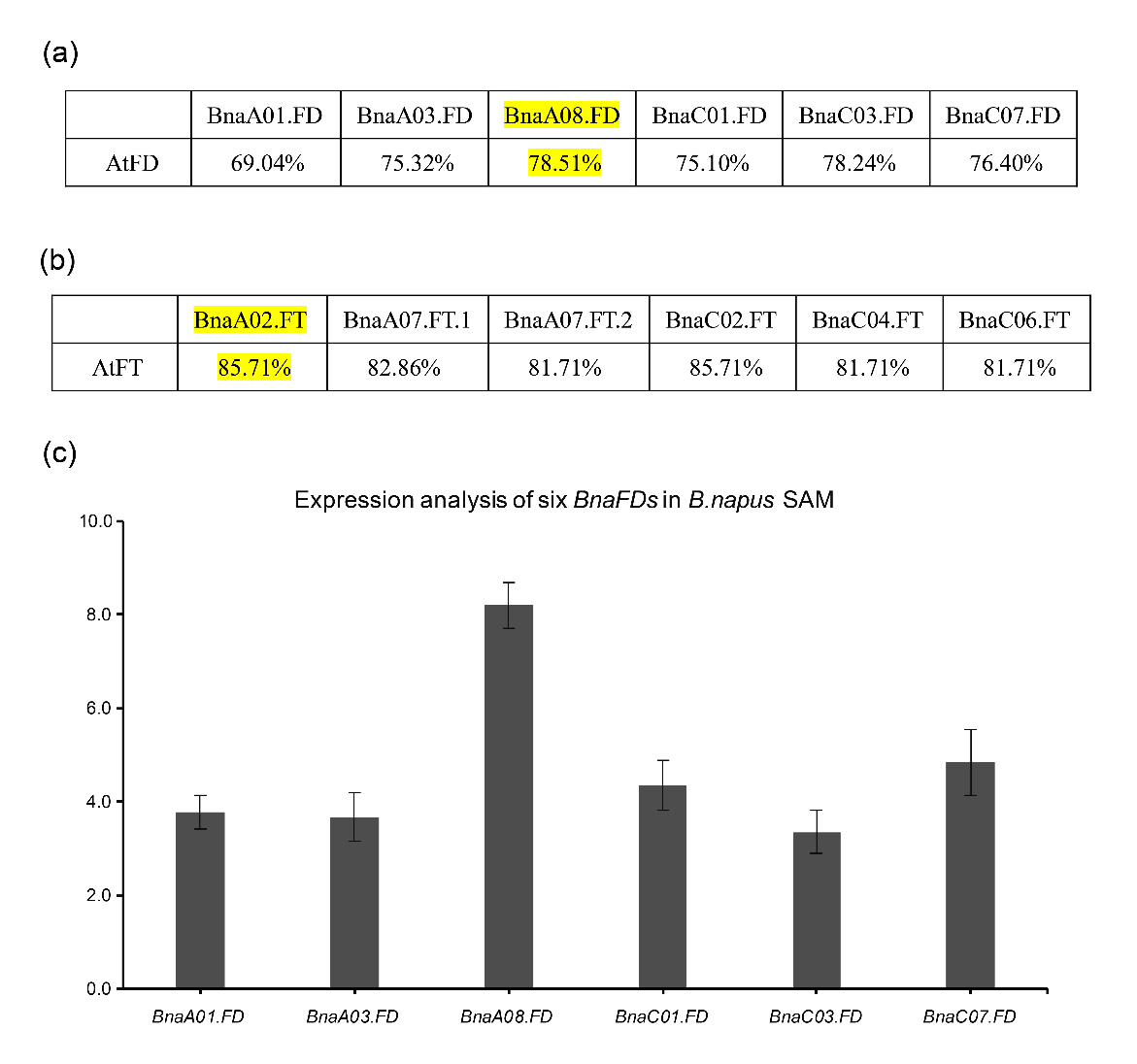


**Fig. S8** *BnaA10/C09.TFL1* participates in multiple regulatory pathways.**(a)** A total of 14,589 (573-DTI vs NIL-573-IDTI) and 4,505 (9 H vs 13 H) differentially expressed genes (DEGs) were identified, with 2,761 DEGs common to both groups. The SAMs from the budding stage of 573-DTI and NIL-573-IDTI at 13 hours, as well as 573-DTI at 9 hours of light treatment, were collected for RNA extraction. **(b, c)** KEGG and GO analysis of the 2,761 overlapping DEGs.


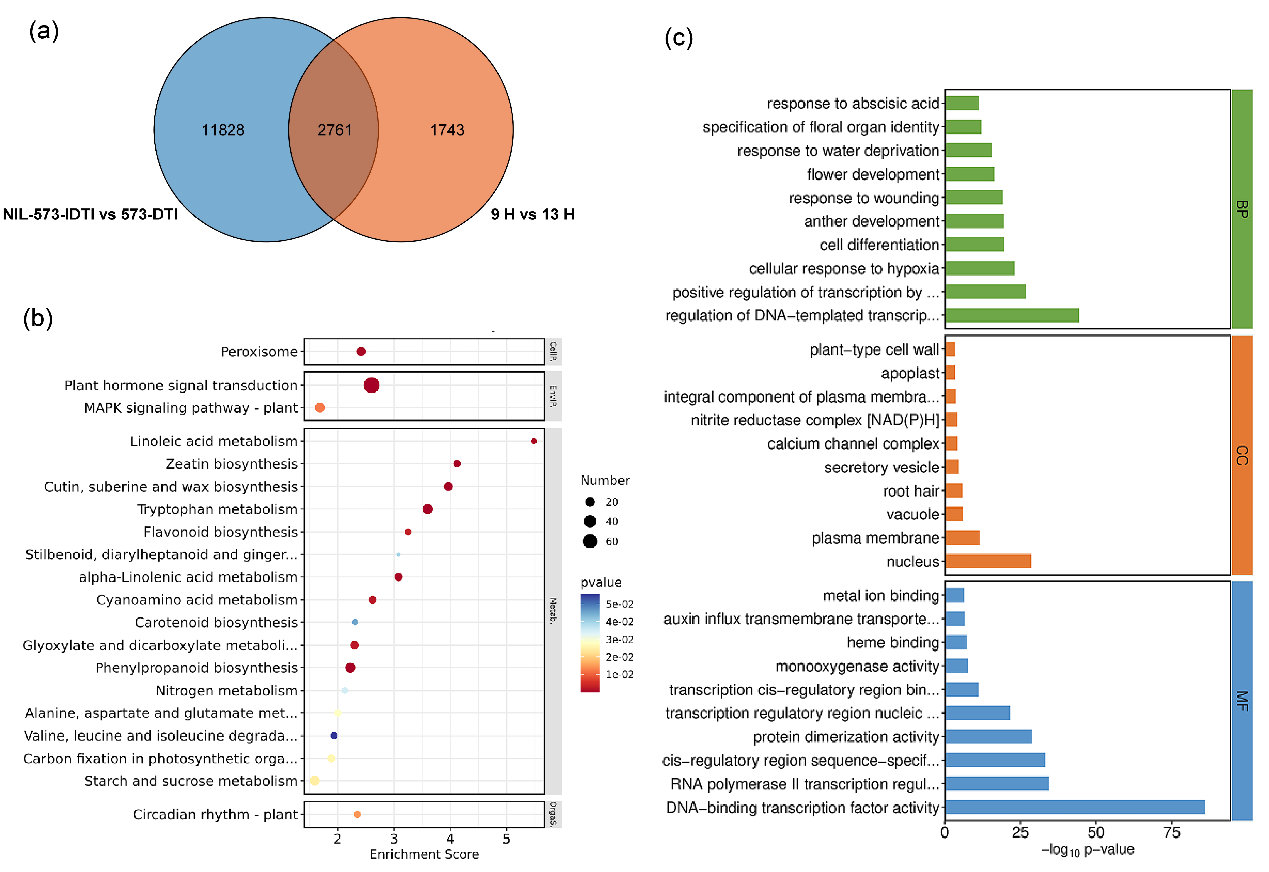


**Fig. S9** Heat map of differentially expressed genes (DEGs) involved in flowering time and floral organ development in the stem apical meristem (SAM) of line 573-DTI and NIL-573-IDTI.The threshold for determining DEGs was defined as |Log2 (fold-change)| >1 and q-value < 0.05.


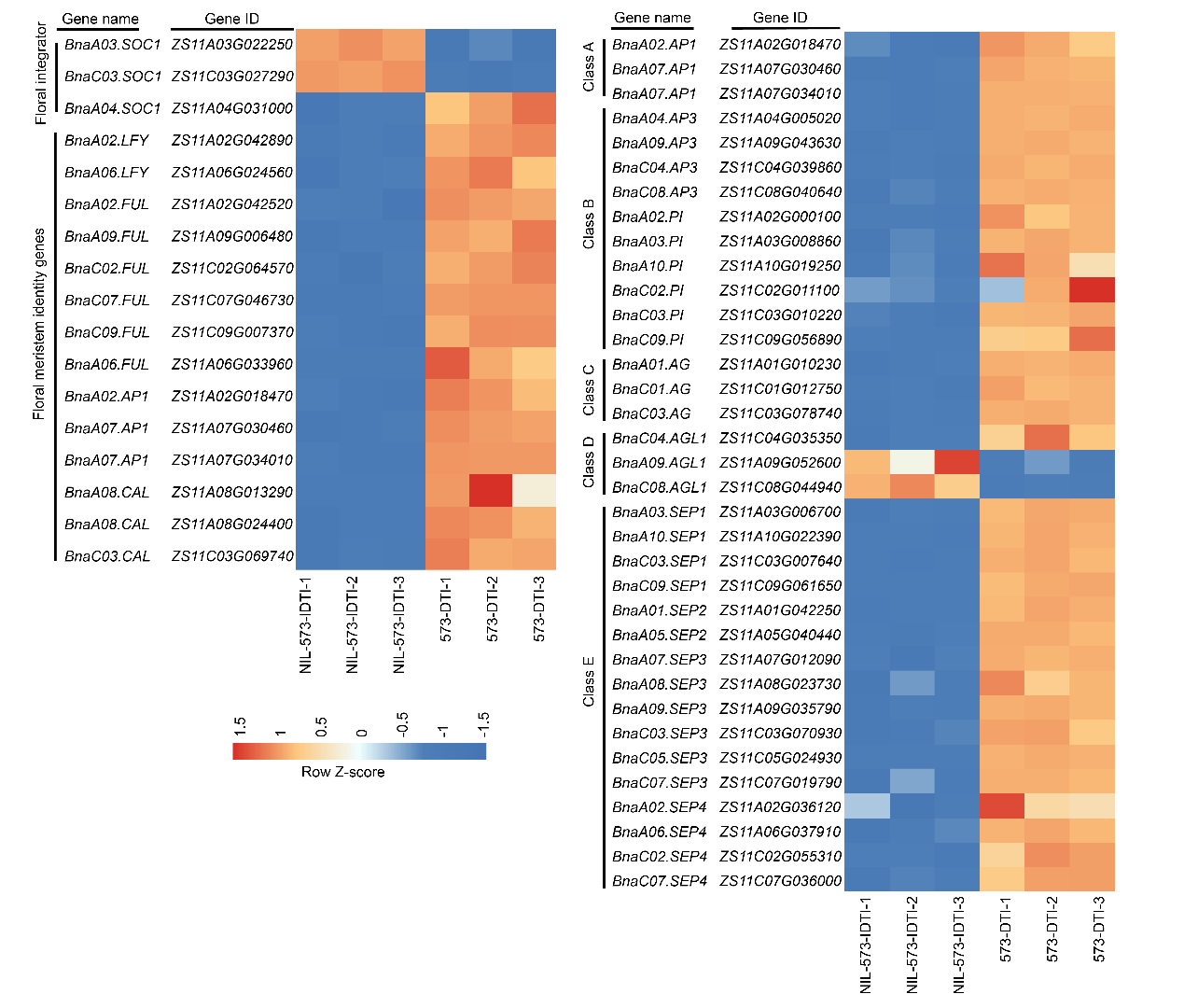


**Fig. S10** Heat map of differentially expressed genes (DEGs) involved in flowering time and floral organ development in the shoot apical meristem (SAM) of line 573-DTI under 9 h and 13 h light duration treatments.The threshold for determining DEGs was defined as |Log2 (fold-change)|＞1 and q-value＜0.05.


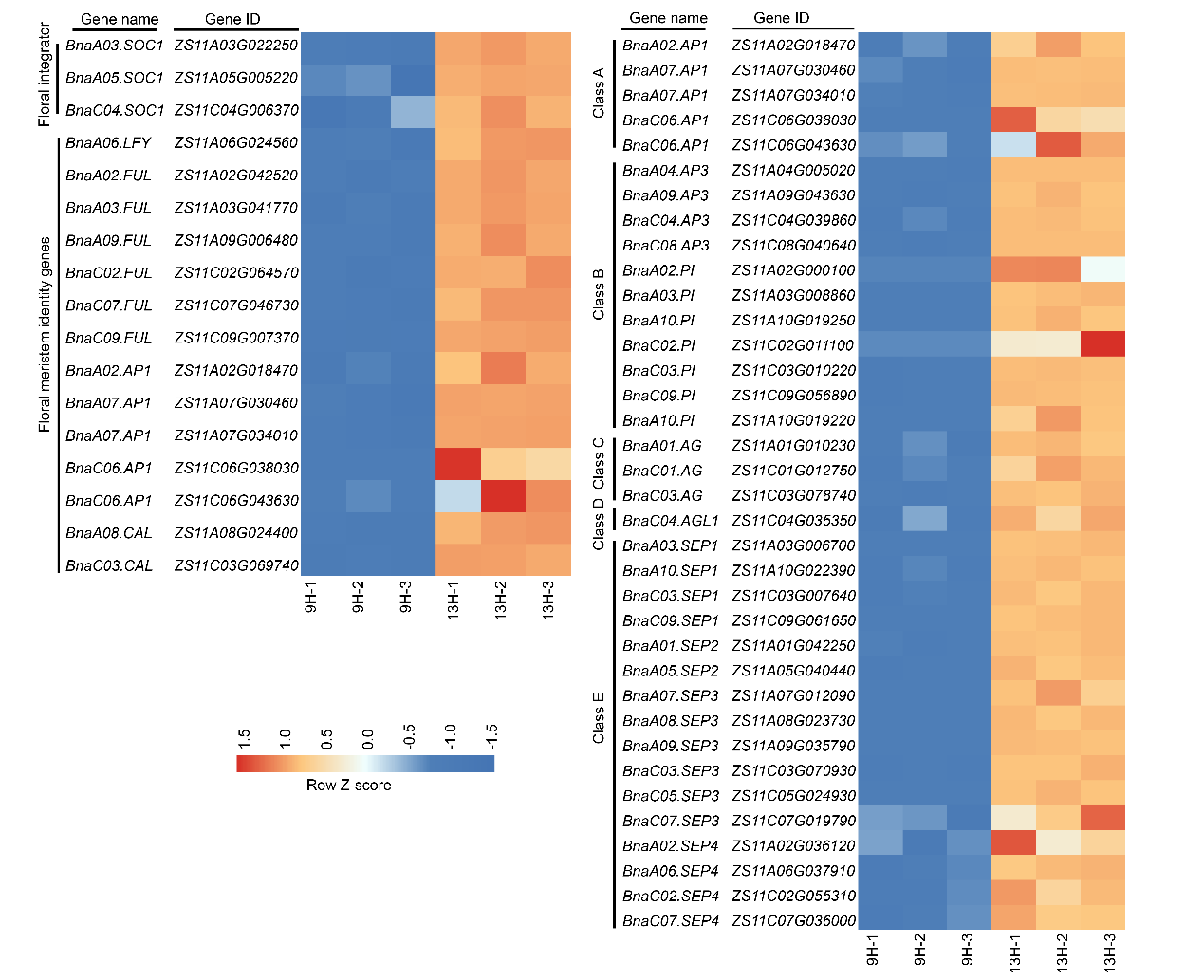

Supplement: Web_Material_uhaf151 [file web_material_uhaf151.zip › supplement figures.docx]
